# Rewarding patient-directed research: Excellence in Translational Medicine Award

**DOI:** 10.1186/1479-5876-4-19

**Published:** 2006-05-03

**Authors:** Christian Brander, Soldano Ferrone, Francesco M Marincola

**Affiliations:** 1 Partner AIDS Research Center, Mass General Hospital and Harvard Medical School, Boston, MA, USA; 2 Department of Immunology, Roswell Park Cancer Institute, Buffalo, NY, USA; 3 Immunogenetics Section, Department of Transfusion Medicine, Clinical Center, National Institutes of Health, Bethesda, Maryland, 20854 –, USA

## Abstract

The Editorial Board of *Journal of Translational Medicine *is pleased to announce a prize to recognize outstanding contributions in the field of translational medicine. The prize is sponsored by Pfizer Global Research and Development, Global Translational Medicine and supported by the Journal of Translational Medicine Editorial Board.

## Rewarding clinically-relevant, evidence-based research

It has been almost three years since we launched the *Journal of Translational Medicine *on July 24th, 2003 [1] as an open access journal devoted to the appropriate peer review and rapid publication of results generated by clinical investigations. The uniqueness of the Journal resides in the major, if not exclusive emphasis on translational medicine. This strategy stems from the realization that we have to satisfy two needs in order for translational medicine to flourish. *First*, it is widely acknowledged that results obtained in animal model systems, although a very valuable source of information, are only rarely super-imposable to those obtained in an clinical setting, even following genetic manipulation of animal models. This realization has provided the impetus to implement clinical investigations in order to assess the clinical relevance of information derived from *in vitro *studies and from animal models. This approach has been possible thanks to the major recent technological progress which has facilitated the application of basic research to address clinically relevant questions in a clinical setting. *Second*, the general realization of the difficulties for journals with emphasis on basic research to accommodate translational medicine papers has provided the rationale for the development of a forum fully dedicated to translational medicine.

The launch and successful development of the *Journal of Translational Medicine *has been possible thanks to the enthusiastic participation of investigators who have submitted manuscripts to the journal and to the active participation of a specialized Editorial Board and investigators with expertise in the clinical and the basic aspects of science.

The journal has received enthusiastic international support and has been rapidly recognized and tracked by PubMed, PubMed Central, Thomson Scientific (ISI), Scopus and Embase. Together with high quality research articles, we have received and published several important contributions [2-11] related to the obstacles and opportunities encountered when applying the emerging discipline of Translational Medicine. We strongly encourage our colleagues to continue this important dialogue since it helps us to sharpen and adjust the directions of the journal to the evolving changes in translational medicine.

To stimulate research in translational medicine and to enhance the submission of high quality manuscripts the journal is establishing a prize, "The Excellence Award in Translational Medicine," with the purpose of rewarding efforts to link science to bedside care. The prize is sponsored by Pfizer Global Research and Development, Global Translational Medicine, and supported by the Journal of Translational Medicine Editorial Board.

Although we recognize the importance and positive impact of commentaries and review articles on the development of translational medicine, only articles including original data will be considered for the award.

## The Excellence in Translational Medicine Award

The recipient of The Excellence in Translational Medicine Award will receive a $2,500 prize to cover the expenses for any meeting sponsored by a non-for-profit organization that is relevant to the goal of translational medicine/research. Submitted articles will be evaluated with regard to: relevance to the purposes of translational medicine/research translating new ideas into patient care (bench to bedside) and/or discovery-driven, evidence-based studies aimed at understanding human pathology by the direct study of human subjects (bedside to bench). Selection criteria will include scientific merit, sound research design, rigorous methodology, originality and clarity.

## Eligibility criteria and selection process

• The award will be granted to the first author of a Research or Methodology manuscript containing original data published in the *Journal of Translational Medicine *between July 1^st ^2006 and June 30^th ^2007.

• Candidates may be self nominated or nominated by a colleague.

• Candidates must be the primary clinician or investigator involved in the project described by the article. If there are multiple first authors, each first author of the selected manuscript may claim designation as a candidate. In the event of multiple authors sharing the award, the prize will be divided equally. If there are multiple first authors, this must be indicated at the time of submission.

• Authors from laboratories of the members of the selection committee are not eligible for the Award.

## Evaluation criteria

Submitted articles will be evaluated with regard to:

• Relevance to the purposes of translational medicine/research translating new ideas into patient care (bench to bedside) and/or discovery-driven, evidence-based studies aimed at the understanding of human pathology by the direct study of human subjects (bedside to bench)

• Scientific merit

• Sound research design

• Rigorous methodology

• Originality

• Clarity

## How to apply

All applications should be made via the online application form. Candidates may be self-nominated, or nominated by others. Nominations must be received between July 1^st ^2006 and June 30^th ^2007.

• Online application form: 

## Selection committee

Selection will be made by a committee of members of the Editorial Board chaired by Prof Richard J. Ablin, University of Arizona, Tucson, AZ, USA and Prof Yi-Xin Zen, Sun Yat-sen University Cancer Center, Guangzhou, China.

We hope the Excellence in Translational Medicine Award will contribute, together with other initiatives, to encourage and reward those investigators who are devoted to improving the efficiency in which biomedical research is performed with the ultimate goal of preventing disease.

## References

[B1] Marincola FM (2003). Translational me: a two way road. J Transl Med.

[B2] Lehmann F, Lacombe D, Therasse P, Eggermont AM (2003). Integration of TranslationalResearch in the European Organization for Research and Treatment of Cancer Research (EORTC) Clinical Trial Cooperative Group Mechanisms. J Transl Med.

[B3] Ioannidis JP (2004). Materializing research promises: opportunities, priorities and conflicts in translational medicine. J Transl Med.

[B4] Mankoff SP, Brander C, Ferrone S, Marincola FM (2004). Lost in translation: obstacles to Translational Medicine. J Transl Med.

[B5] Parks MR, Disis ML (2004). Conflicts of interest in translational research. J Transl Med.

[B6] Webb CP, Pass HI (2004). Translation research: from accurate diagnosis to appropriate treatment. J Transl Med.

[B7] Carter RE, Woolson RF (2004). Statistical design considerations for pilot studies transitioning therapies from the bench to the bedside. J Transl Med.

[B8] Horig H, Pullman W (2004). From bench to clinic and back: Perspective on the 1st IQPC Translational Research conference. J Transl Med.

[B9] Joiner KA (2005). The not-for-profit form and translational research: Kerr revisited?. J Transl Med.

[B10] Sonntag KC (2005). Implementations of translational medicine. J Transl Med.

[B11] Marincola E (2006). Why is public science education important?. J Transl Med.

